# Comparison of ex vivo bioluminescence imaging, *Alu*-qPCR and histology for the quantification of spontaneous lung and bone metastases in subcutaneous xenograft mouse models

**DOI:** 10.1007/s10585-024-10268-4

**Published:** 2024-02-14

**Authors:** Marie-Therese Haider, Vera Freytag, Linda Krause, Tanja Spethmann, Tobias Gosau, Mia C. Beine, Christine Knies, Jennifer Schröder-Schwarz, Michael Horn, Kristoffer Riecken, Tobias Lange

**Affiliations:** 1 Institute of Anatomy and Experimental Morphology, University Medical Center Hamburg- Eppendorf, Martinistrasse 52, Hamburg, 20246 Germany; 2 Institute of Medical Biometry and Epidemiology, University Medical Center Hamburg-Eppendorf, Hamburg, Germany; 3 Core Facility In Vivo Optical Imaging, University Cancer Center Hamburg, University Medical Center Hamburg-Eppendorf, Hamburg, Germany; 4 Mildred Scheel Cancer Career Center, University Medical Center Hamburg-Eppendorf, Hamburg, Germany; 5 Research Department Cell and Gene Therapy, Department of Stem Cell Transplantation, University Medical Center Hamburg-Eppendorf, Hamburg, Germany; 6 Institute of Anatomy I, University Hospital Jena, Teichgraben 7, Jena, 07743 Germany; 7 Comprehensive Cancer Center Central Germany (CCCG), Ulm, Germany

**Keywords:** Bone metastases, Ex vivo BLI, Alu-qPCR, Immunohistochemistry

## Abstract

Bioluminescence imaging (BLI) is a non-invasive state-of-the-art-method for longitudinal tracking of tumor cells in mice. The technique is commonly used to determine bone metastatic burden in vivo and also suitable ex vivo to detect even smallest bone micro-metastases in spontaneous metastasis xenograft models. However, it is unclear to which extent ex vivo BLI correlates with alternative methods for metastasis quantification. Here, we compared ex vivo BLI, human DNA-based *Alu*-qPCR, and histology for the quantification of bone vs. lung metastases, which are amongst the most common sites of metastasis in prostate cancer (PCa) patients and spontaneous PCa xenograft models. Data from 93 immunodeficient mice were considered, each of which were subcutaneously injected with luciferase/RGB-labeled human PCa PC-3 cells. The primary tumors were resected at ~ 0.75 cm³ and mice were sacrificed ~ 3 weeks after surgery and immediately examined by ex vivo BLI. Afterwards, the right lungs and hind limbs with the higher BLI signal (BLI^Hi^ bone) were processed for histology, whereas the left lung lobes and hind limbs with the lower BLI signal (BLI^Lo^ bone) were prepared for *Alu*-qPCR. Our data demonstrate remarkable differences in the correlation coefficients of the different methods for lung metastasis detection (*r* ~ 0.8) vs. bone metastasis detection (*r* ~ 0.4). However, the BLI values of the BLI^Hi^ and BLI^Lo^ bones correlated very strongly (*r* ~ 0.9), indicating that the method per se was reliable under identical limitations; the overall level of metastasis to contralateral bones was astonishingly similar. Instead, the level of lung metastasis only weakly to moderately correlated with the level of bone metastasis formation. Summarized, we observed a considerable discrepancy between ex vivo BLI and histology/*Alu*-qPCR in the quantification of bone metastases, which was not observed in the case of lung metastases. Future studies using ex vivo BLI for bone metastasis quantification should combine multiple methods to accurately determine metastatic load in bone samples.

**Supplementary Information:**

The online version contains supplementary material available at 10.1007/s10585-024-10268-4.

## Introduction

In 2022, prostate cancer (PCa) was the most commonly diagnosed cancer in men [1, 2] and after lung cancer it also accounted for the greatest number of cancer-related deaths in male patients [1, 2]. Importantly, the incidence of patients with distant/ metastatic prostate cancer increased by 6% over the last decade [2]. At this stage, the disease remains incurable and the 5-year relative survival drops from over 99% to only 30% when compared to patients with localized or regional disease [2]. In PCa the majority of patients with advanced disease (> 80%) develop bone metastases [3].

Due to the complexity of the metastatic cascade, metastasis research mainly relies on the use of animal models [4]. Our long-standing expertise is the development of subcutaneous (s.c.) xenograft models of solid human cancers in immunodeficient mice [5–8]. Such xenograft models have the advantage that tumor cells previously cultured in vitro must first adapt to the environmental conditions within an establishing primary tumor (i.e.: three-dimensional organization, tumor-stroma interaction, cancer-associated fibroblasts and macrophages, hypoxia, etc.), before they start to metastasize spontaneously. During this adaptation process, individual primary tumor cells or clusters undergo phenotypic switches such as epithelial-mesenchymal transition (EMT) which is thought to promote anoikis suppression and hence survival of future metastatic cells in the bloodstream; and its reversal, i.e. mesenchymal-epithelial transition (MET), as a putative requirement for dormancy suppression and metastatic outgrowth [9]. In addition, pre-metastatic niche formation takes place in spontaneous metastasis models through primary tumor-released factors [10], which also crucially determines the fate of metastasizing cells in a site-specific manner [11]. One of the disadvantages of spontaneous metastasis models is the usually low incidence of developing metastases and the necessary proof of human origin of metastatic cells especially for small, routine histologically doubtful cell clusters and single disseminated tumor cells (DTCs).

The labeling of tumor cells with luciferase (Luc, Luc2) and subsequent bioluminescence imaging (BLI) is a widely accepted, simple method for detecting the accumulation of metastatic cells throughout the organism of experimental animals [12]. In particular, in the field of bone metastasis research, BLI is commonly used in an in vivo approach and serves as a direct measure of the longitudinal growth of bone metastases, mainly after intracardiac or intracaudal artery injection of large tumor cell counts [13], and sometimes even without validation by further methods such as histology-based quantification. In our previous studies using s.c., spontaneously metastatic xenograft models, we have shown that BLI is also suitable in an ex vivo approach shortly after sacrifice of animals to detect even smallest micro-metastases in isolated bones by histology [10, 14, 15]. In addition, immunohistochemistry (IHC) using human-specific antibodies or antibodies against firefly luciferase is feasible to proof the human origin of such metastatic deposits [10, 14].

However, our cumulative experience with the detection of bone metastases by ex vivo BLI suggested that in a notable percentage of mice the ex vivo BLI signals could not be validated by histology while in some cases large, vital bone metastases as per histology were not apparent via ex vivo BLI. One further technique we have been using regularly in parallel for the quantification of the bone metastatic burden, is quantitative real time-PCR for human-specific DNA sequences (*Alu*-qPCR) [7, 14, 16, 17]. Therefore, the aim of the present study was to compare ex vivo BLI, *Alu*-qPCR and histology as three independent methods for the quantification of spontaneous metastases in xenograft models, specifically comparing their value for lung and bone metastasis quantification.

## Materials and methods

### Cell culture

Human prostate cancer PC-3 cells (obtained from ATCC, CRL-1435) were cultivated and transduced with lentiviral Luc2-containing RGB vectors as previously described [18].

### Animal experiments

**Fig. 1:**
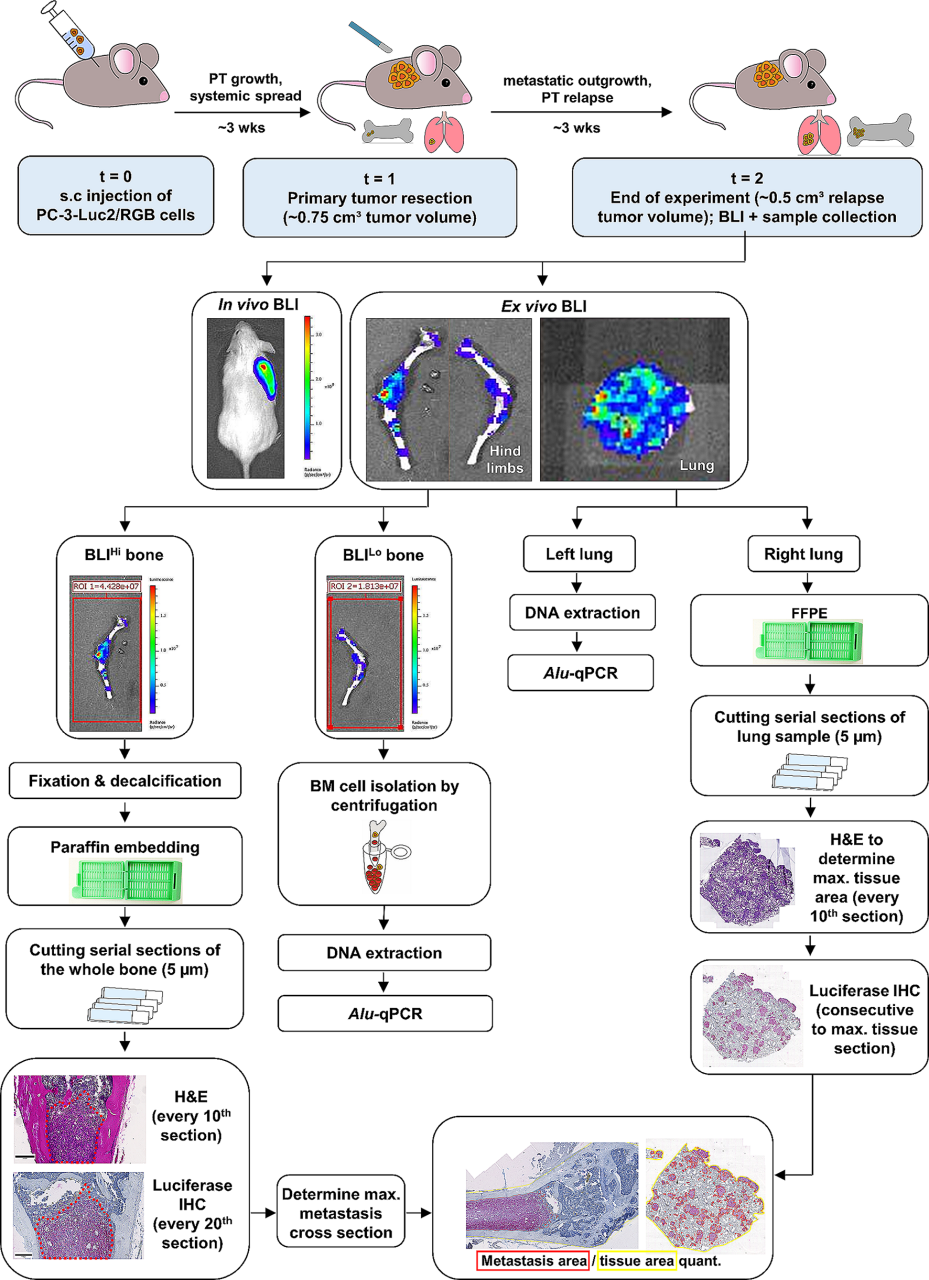
Schematic study design. 8 to 12 weeks old mice (total *n* = 93) were subcutaneously (s.c.) injected above the scapula with 1 × 10^6^ tumor cells. The subcutaneous tumors were allowed to grow and surgically removed at a size of ~ 0.75 cm³. Postoperatively, mice were followed up for about 3 weeks. In vivo bioluminescence imaging (BLI) of relapsing tumors at the primary site and ex vivo BLI of isolated hind limbs and organs was performed. Metastatic burden in long bones was further assessed using immunohistochemistry (BLI^Hi^) and human-specific *Alu*-qPCR (BLI^Lo^). Similarly, metastatic burden in lung samples was determined using *Alu*-qPCR (left lung lobe) and histology (right lung lobe)

Within the course of different projects, 8 to 12 weeks old mice (total *n* = 93) with an NSG background (NOD.Cg-Prkdc^scid^ Il2rg^tm1Wjl^/SzJ; Jax, Stock 005557) were subcutaneously (s.c.) injected above the scapula with 1 × 10^6^ tumor cells in 200 µL cell culture media without supplements. The subcutaneous tumors were allowed to grow and surgically removed at a size of ~ 0.75 cm^3^ as described [14]. Postoperatively, mice were followed up for about 3 weeks. During this period, all mice eventually suffered from primary tumor relapse. At a primary tumor relapse size of ~ 0.75 cm³, the mice were finally anesthetized with ketamine/xylazine and sacrificed by cervical dislocation. All animal experiments were approved by the local authorities (project number: N109/2019, N017/2020, N025/2021; Behörde für Justiz und Verbraucherschutz der Freien und Hansestadt Hamburg, Lebensmittelsicherheit und Veterinärwesen, Lebensmittelsicherheit und Veterinärwesen) and all methods were performed in accordance with the relevant guidelines and regulations. This study is reported in accordance with the ARRIVE guidelines. The experimental setup for the generation of spontaneous lung and bone metastases using s.c. xenograft mouse models and their quantification by ex vivo BLI, *Alu*-qPCR and histology is summarized in Fig. 1. The numbers of mice with available data from the respective quantification methods are provided in Table 1.

**Table 1:** Presence of spontaneous metastases in lung and bone assessed by Alu-qPCR, histology and bioluminescence imaging (BLI)

Method	Organ	Metastasis detected	No Metastasis detected
*Alu-*qPCR	Lung	100% (*n* = 93)	0% (*n* = 0)
Histology	Lung	100% (*n* = 13)	0% (*n* = 0)
BLI	Lung	5.72 × 10^6^ p/s to 4.82 × 10^10^ (*n* = 91)	
*Alu-*qPCR	Bone	71.62% (*n* = 53)	28.38% (*n* = 21)
Histology	Bone	18.67% (*n* = 14)	81.33% (*n* = 61)
BLI	Bone	BLI^Hi^ 1.69 × 10^5^ p/s to 2.02 × 10^10^ p/s BLI^Lo^ 8.61 × 10^4^ p/s to 8.61 × 10^6^ p/s	(*n* = 75)

### Bioluminescence Imaging (BLI)

At the time of sacrifice, mice were intraperitoneally (i.p.) injected with 150 mg/kg body weight luciferin (Sigma, Steinheim, Germany). Ten minutes after luciferin injection, mice were scanned by in vivo BLI (IVIS 200, Perkin Elmer, Waltham, MA, USA) using the auto-exposure setting to detect functional luciferase expression in the relapsing primary tumors. Immediately thereafter, the mice were sacrificed, lungs or hind limbs isolated and individually re-scanned ex vivo. From the hind limbs, all surrounding muscle tissue was removed using scalpel, scissors and cellulose cloths prior to the ex vivo re-scan. The BLI signal intensities are presented as total flux (photons per second, [p/s]) and were quantified with Living Image Software (Perkin Elmer). The precision of BLI to detect PC-3 RGB luc2 cells was previously validated in vitro (Supplemental Fig. 1A).

### Sample collection and processing

#### Alu-qPCR-based quantification of spontaneous metastases

The left lungs or hind limbs with the lower BLI signal (BLI^Lo^ bones) were further processed for *Alu*-qPCR-based quantification of metastatic cell loads. Importantly, Alu primers are human specific and do not detect murine DNA resulting in a reliable method of human tumor cell quantification. Lung samples were homogenized in a sample disruptor (Tissue Lyser II, Qiagen); femora and tibiae of BLI^Lo^ hind limbs were cut transversely in the middle of the diaphysis and placed cut-side down in bottom-opened PCR reaction tubes, which were inserted into 1.5 mL Eppendorf tubes containing 500 µl PBS. These tubes were centrifuged for 1 min at 1500 x g. The resulting bone marrow pellets as well as the lung homogenates were subjected to DNA isolation using the peqGOLD Tissue DNA Mini Kit according to the manufacturer’s instructions (VWR). DNA concentrations were quantified using a NanoDrop spectrophotometer (Peqlab) and normalized to a concentration of 60 ng/µL DNA in all samples using elution buffer from the isolation kit. Finally, 2 µL total DNA of each sample were used in the subsequent *Alu*-qPCR using established primers (Forward: TGG CTC ACG CCT GTA ATC CCA Reverse: GCC ACT ACG CCC GGC TAA TTT) and protocols (LightCycler 480, Roche) [19]. The number of human cells per DNA template was calculated with a standard curve generated by diluting the DNA of 2 × 10^3^ to 2 × 10^− 3^ PC-3-Luc2/RGB cells (Supplemental Fig. 1B). Lung or bone marrow DNA extracts from healthy, tumor-free control mice of the same mouse strain served as negative controls (background DNA, no human DNA added). Ct values ≥ those of the background DNA were manually set to ‘negative’ indicating that no tumor cells could be detected.

### Histology-based quantification of spontaneous metastases

After resection of the lungs and hind limbs, the s.c. xenograft primary tumors were harvested, fixed in 3.7% formalin in 0.1 M phosphate-buffered saline for 24 h (hrs), and embedded in paraffin wax for subsequent sectioning and anti-luciferase immunohistochemistry (IHC; see below). Right lungs were fixed in 3.7% formaldehyde in 0.1 M phosphate-buffered saline for 24 h at 4 °C. Lungs were then cut into 1-mm-thick pieces, and all sections were brought into a plane by gently pressing them into pre-warmed agar with a syringe plunger. After hardening of the agar, the lung tissues were dehydrated and embedded in paraffin wax. Five µm thick sections were cut and every 10th section hematoxylin and eosin (H&E)-stained as previously described [20]. Further sections were cut for IHC. The H&E section with the maximum amount of visible lung tissue was determined for each mouse using a light microscope (Axioskop, Zeiss, Jena, Germany), and the consecutive section stained anti-luciferase (see below). This luciferase-stained section was digitized (Z1 AxioScan, Zeiss), the areas [µm²] of all luciferase-positive metastases visible on that slide were quantified, summed, and this sum was normalized to the total lung tissue area [µm²] on the respective slide (ZEN 3.2 software (blue edition); Zeiss).

The BLI^Hi^ bones were fixed in 3.7% formaldehyde in 0.1 M phosphate-buffered saline for 24 h at 4 °C, then decalcified in 10% EDTA for 48 h, dehydrated, embedded in paraffin wax and the whole bone cut into 5 μm thick sections. Every 10th section was H&E stained for visual control of the respective section depth (gating of the bone marrow cavity, presence of bone marrow) and every 20th section was later used for IHC against luciferase (see below). All luciferase-stained sections were examined for the presence of luciferase-positive metastases or single cells (Supplemental Fig. 1C) using a light microscope (Axioskop, Zeiss) and the cross section with the maximum visible metastasis area was digitized (Z1 AxioScan, Zeiss). The luciferase-positive metastasis area [µm²] was determined on this slide and normalized to the metastasis bearing bone tissue area [µm²] on the same slide (ZEN 3.2 software (blue edition); Zeiss), and depicted as metastasis area/bone area [%]. Additionally, we estimated the metastasis volumes [µm³] by calculating the average bone metastasis areas of all quantified sections per bone, multiplied by the total tissue depth (every 20th slide was analyzed, if a metastasis was observed on slide 20 and 40, the total depth was 100 μm as each section had a thickness of 5 μm). The Ki67^+^ metastasis area [%] was measured by interactively drawing around the Ki67^+^ tumor cells using Fiji (ImageJ) software and finally normalized to the total metastasis area (Fig. 5A).

### Immunohistochemistry (IHC)

Lung and bone metastases were visualized and characterized using anti-luciferase and anti-Ki67 IHC. Anti-luciferase IHC was performed as described [15]. Briefly, lung and bone sections were deparaffinized, pretreated in citrate buffer at pH 6 for either 20 min in a steamer at 100 °C (in the case of lung sections) or overnight in a water bath at 60 °C (in the case of bone sections) for Luciferase IHC. For Ki67 IHC samples were incubated in citrate buffer at pH 6 in a water bath at 85 °C overnight. After washing, sections were incubated with primary antibodies (anti-firefly luciferase (#ab181640, abcam, Cambridge, UK; 2 µg/mL) or anti-Ki67 (Ki67 Mib 1, DAKO, 1.1 µg/mL)) for 1 h at room temperature. After washing, sections were incubated with a biotinylated rabbit-anti-goat secondary antibody (#E0466, Dako, Agilent, Santa Clara, CA, USA; diluted 1:200, 30 min, room temperature) or goat Anti‑Mouse IgG secondary antibody (LS‑C149505, LS Bio, LS-C149505). After washing, antibody binding was detected using a streptavidin-alkaline phosphatase kit (ABC-AP, Vector Labs., Peterborough, UK) and visualized using liquid permanent red (Dako). Nuclei were counterstained using Mayer’s hemalum solution for 5 to 10 s. Stained samples were sealed with coverslips in an aqueous mounting agent (Aquatex, Sigma). Of note, Ki67 stained sections of long bones were not dehydrated prior to coverslipping. The IHC protocols were established on sections of the corresponding xenograft primary tumors, with nonspecific staining reactions controlled by the use of appropriate isotype controls (polyclonal goat Ig).

### Statistics

Correlation between lung and bone metastasis values as determined by ex vivo BLI (total photon flux per second [p/s]), *Alu*-qPCR (number of tumor cells per 60 ng lung or bone marrow DNA) and histology (metastasis area [µm²] per tissue area [µm²] in %, metastasis volume [µm^3^]) was quantified with Spearman’s rho statistic. Corresponding 95% confidence intervals were calculated by bootstrapping. Bland Altman analyses were performed on log-transformed (logarithm to basis 10) data where 0.1 was added to measurements of size zero before log-transformation and using the “BlandAltmanLeh” package in R (version 0.3.1, cite: Bernhard Lehnert (2015). BlandAltmanLeh: Plots (Slightly Extended) Bland-Altman Plots. R package version 0.3.1. https://CRAN.R-project.org/package=BlandAltmanLeh). Graphs were plotted with GraphPad Prism 9.3.1 (2022 GraphPad software). Statistical analysis was performed with R version 4.1.2. The correlation between two variables was interpreted as follows: negligible correlation if *r* = 0.00–0.10, weak correlation if *r* = 0.10–0.39, moderate correlation if *r* = 0.40–0.69, strong correlation if *r* = 0.70–0.89 and very strong correlation *r* = 0.90–1.00) [21].

## Results

### Correlation analysis for *Alu*-qPCR, histology and ex vivo BLI in lung samples

To determine metastatic load in lung samples of our spontaneous metastasis xenograft models we performed ex vivo BLI of the whole lung, human specific *Alu*-qPCR of the left lung lobe, and histological analysis of luciferase-stained lung metastases in one representative section of the right lung lobe.

**Fig. 2:**
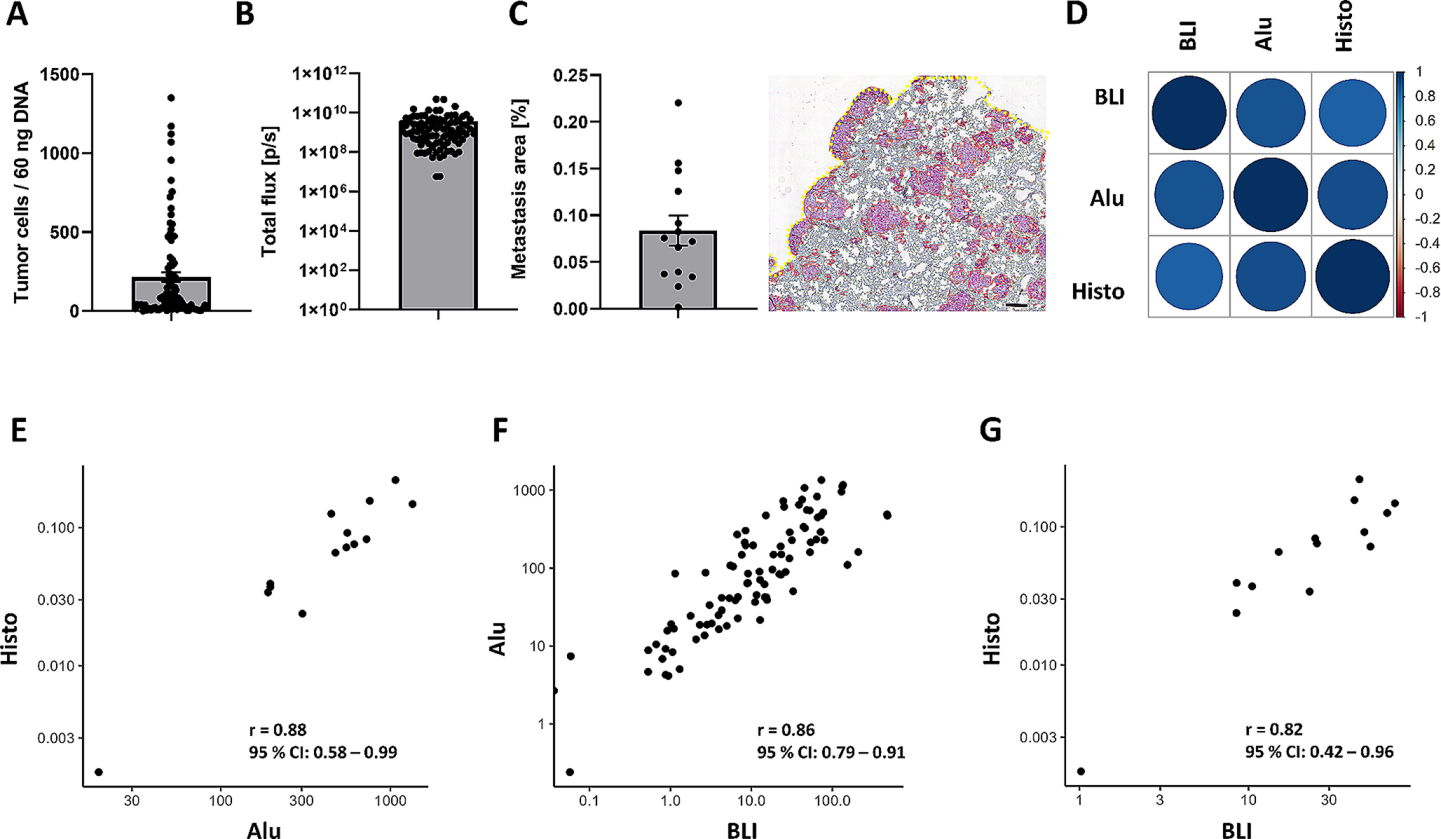
Correlation analysis for spontaneous lung metastases. Development of lung metastasis in the spontaneous xenograft model was determined using **A***Alu*-qPCR (Alu, depicted as DNA of human tumor cells/60 ng lung DNA), **B** ex vivo bioluminescence imaging (BLI, depicted as photon flux per second (p/sec)) and **C** histological assessment (Histo, depicted as metastasis area per lung area [%]). A representative lung section after anti-luciferase staining is shown in (**C**). Luciferase-positive tumor cells are shown in pink, metastasis areas are highlighted in red, tissue area in yellow; scale bar is 200 μm. **D** Correlation analysis plot for the three methods with **E** Histo vs. Alu, **F** Alu vs. ex vivo BLI and **G** Histo vs. ex vivo BLI. Correlation was quantified with Spearman’s rho statistic. *n* = 92/group for BLI and Alu, *n* = 14 for Histo

Importantly, all three methods were able to detect spontaneously metastasized tumor cells in lung samples. *Alu*-qPCR detected human tumor cell DNA in 100% of analyzed mice. Values ranged from as little as 0.241 to 1,350 cells/60 ng DNA (Fig. 2A; Table 1). Ex vivo BLI signals ranged from 5.72 × 10^6^ p/s up to 4.82 × 10^10^ p/s (Fig. 2B) and histological analysis revealed a metastasis area of 0.002–0.22% (Fig. 2C).

Overall, there was a strong positive correlation between all three methods in lung samples (Fig. 2D). The strongest correlation was observed when comparing *Alu*-qPCR vs. histological analysis (Fig. 2E) followed by *Alu*-qPCR vs. ex vivo BLI (Fig. 2F) and ultimately histological analysis vs. ex vivo BLI (Fig. 2G). Thus, histological quantification correlated strongly with the two other methods in case of lung metastases despite a relatively low number of samples (*n* = 13).

### Correlation analysis for *Alu*-qPCR, histology and ex vivo BLI in bone samples

Next, we aimed to assess whether there is a correlation between ex vivo BLI and *Alu*-qPCR (considering bones with relatively weak BLI signal) or histology (considering bones with relatively high BLI signal) for the quantification of spontaneous PCa bone metastases.

**Fig. 3:**
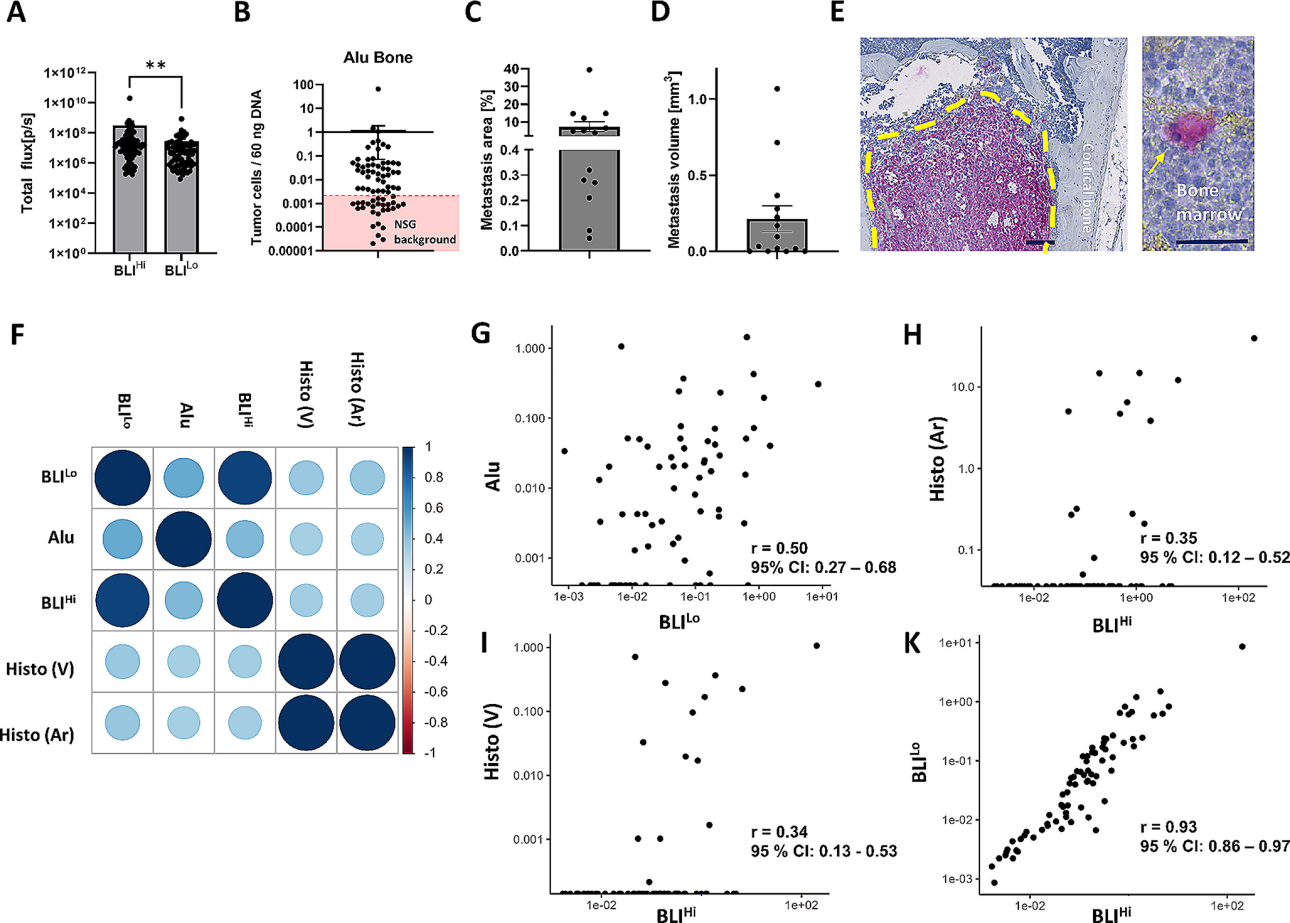
Correlation analysis for spontaneous bone metastases. The presence of spontaneous bone metastasis in the xenograft mouse model was first assessed using **A** bioluminescence imaging (BLI). Left and right hind limbs were imaged ex vivo. The leg with the lower BLI signal (BLI^Lo^) was consequently used for **B***Alu*-qPCR (Alu). Values below the NSG background (red shaded area) were considered as 0 which equals the absence of tumor cells in the bone marrow. The leg with the higher BLI signal (BLI^Hi^) underwent histological assessment; metastatic load was quantified by assessing **C** the metastatic area/bone tissue area (Histo (Ar)) in % and **D** bone metastasis volume (Histo (V)) in µm^3^. **E** shows a histological section of luciferase-positive tumor cells with macro-metastasis being highlighted by yellow dotted line and a single DTC in the bone marrow being pointed out by the yellow arrow. Scale bars indicate 200 μm and 50 μm, respectively. Correlation analysis plot for all methods in **F** with **G** Alu vs. BLI^Lo^, **H** Histo (Ar) vs. BLI^HI^, **I** Histo (V) vs. BLI^HI^ and **J** BLI^Lo^ vs. BLI^HI^. Wilcoxon matched-pairs signed rank test for BLI^Hi^ vs. BLI^Lo^ with ** indicating *p* ≤ 0.01. Correlation was quantified with Spearman’s rho statistic. *n* = 75 for BLI^Lo^, *n* = 73 for Alu, *n* = 75 for BLI^Hi^, Histo (V) and Histo (Ar)

Ex vivo BLI signals measured as total photon flux ranged from 1.69 × 10^5^ p/s to 2.02 × 10^10^ p/s in the BLI^Hi^ and from 8.61 × 10^4^ p/s to 8.61 × 10^6^ p/s in the BLI^Lo^ bone (Fig. 3A; Table 1). *Alu*-qPCR detected smallest fractions of human tumor cell DNA with values ranging from 0.001 up to as many as 65.30 tumor cells/60 ng bone marrow DNA (Fig. 3B). In total, *Alu*-qPCR could detect tumor cells in the bone marrow of 71.62% (*n* = 53) of analyzed animals (total *n* = 74, Table 1). In 28.38% (*n* = 21 Fig. 3B; Table 1) of mice the detected signal was below the NSG-threshold indicating the absence of tumor cells. With regards to histological assessment, we quantified both the total volume of the bone metastasis (mm^3^) as well as the bone metastasis area (%) on the section with the maximum amount of visible bone metastasis which was consequently normalized to the total bone area (Fig. 1). The smallest bone metastasis area/tissue area was 0.05% and the largest 39.45% (Fig. 3C). With regards to the bone metastasis volume, values ranged from 0.0002 mm^3^ to 1.07 mm^3^ (Fig. 3D). Importantly, luciferase-positive tumor cells - either as single DTCs, micro- or macro-metastases (Fig. 3E) - could only be identified in 18.67% of mice by histology (*n* = 14 out of 75, Table 1). Expectedly, the two methods (metastasis area vs. metastasis volume) for histological quantification very strongly correlated positively with each other (*r* = 1.00, 95% CI: 0.99–1.00, Fig. 3F).

We performed ex vivo BLI of both hind limbs prior to *Alu*-qPCR (BLI^Lo^) and histological (BLI^Hi^) analysis (Fig. 1). The strongest correlation between the different methods was observed for *Alu*-qPCR and ex vivo BLI from the corresponding leg (BLI^Lo^), indicating a moderate positive, but relevant correlation (Fig. 3F, G). Surprisingly, we did only observe weak positive correlation for ex vivo BLI analysis and histological analysis of the corresponding leg (Fig. 3F, H and I). Thus, the correlation between the three quantification methods for bone metastases was not as profound as for lung metastases (Fig. 2E–G).

Nevertheless, there was a very strong positive correlation between BLI-analysis of both hind limbs (BLI^Lo^ vs. BLI^Hi^) (Fig. 3F and J).

**Fig. 4:**
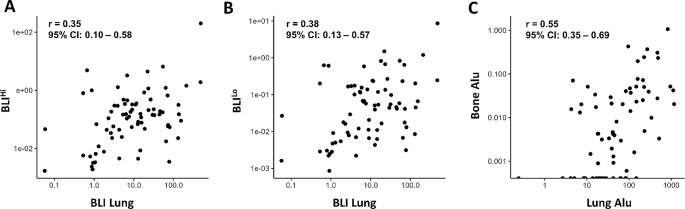
Correlation analysis between the methods for metastasis detection in lung and bone. The presence of spontaneous metastases in lung and bone was assessed using ex vivo BLI and *Alu*-qPCR. Correlation between metastasis detection in lung and bone via ex vivo BLI is shown in (**A**) for BLI^Hi^ bone vs. Lung as well as in (**B**) for BLI^Lo^ bone vs. lung (*n* = 75). Correlation for *Alu*-qPCR is shown in (**C**) with *n* = 73

To additionally assess whether spontaneous metastases occur systemically or rather in a site-specific manner, we investigated the correlation between spontaneous lung and bone metastasis values, also considering whether such putative correlation differs between the respective detection methods (*Alu*-qPCR, BLI). We determined a weak correlation (when comparing ex vivo BLI for the detection of spontaneous metastasis in lung vs. bone (Fig. 4A, B). For *Alu*-qPCR a moderate positive correlation coefficient of 0.55 was observed.

### Bland altman analysis

**Fig. 5:**
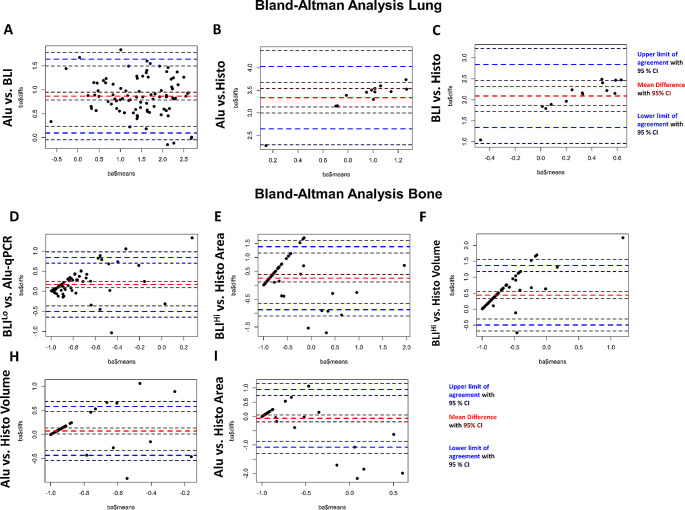
Bland-Altman analysis of methods for the quantification of spontaneous lung and bone metastases. Bland-Altman analysis for the comparability of *Alu-*qPCR (Alu), ex vivo Bioluminescence Imaging (BLI) and histological assessment (Histo) for the detection of spontaneous lung and bone metastases. **A** Alu vs. BLI (*n* = 91) **B** Alu vs. Histo (*n* = 14) and **C** BLI vs. Histo (*n* = 14) for lung metastasis. Bioluminescence Imaging (BLI) and histological assessment (Histo) for the detection of spontaneous bone metastases. BLI^Lo^ vs. Alu is shown in (D, *n* = 71), BLI^Hi^ vs. Histo Area in (E, *n* = 75), BLI^Hi^ vs. Histo Volume in (F, *n* = 75), Alu vs. Histo Volume in (H, *n* = 71) and Alu vs. Histo Area in (I, *n* = 71). Mean of measurements shown on the X-axis and difference between the means shown on the Y-axis; mean is shown as light-red dotted line with 95% confidence interval (CI) as dark-red dotted line; upper and lower agreement are shown as blue dotted line with 95% CI in dark-blue

**Fig. 6:**
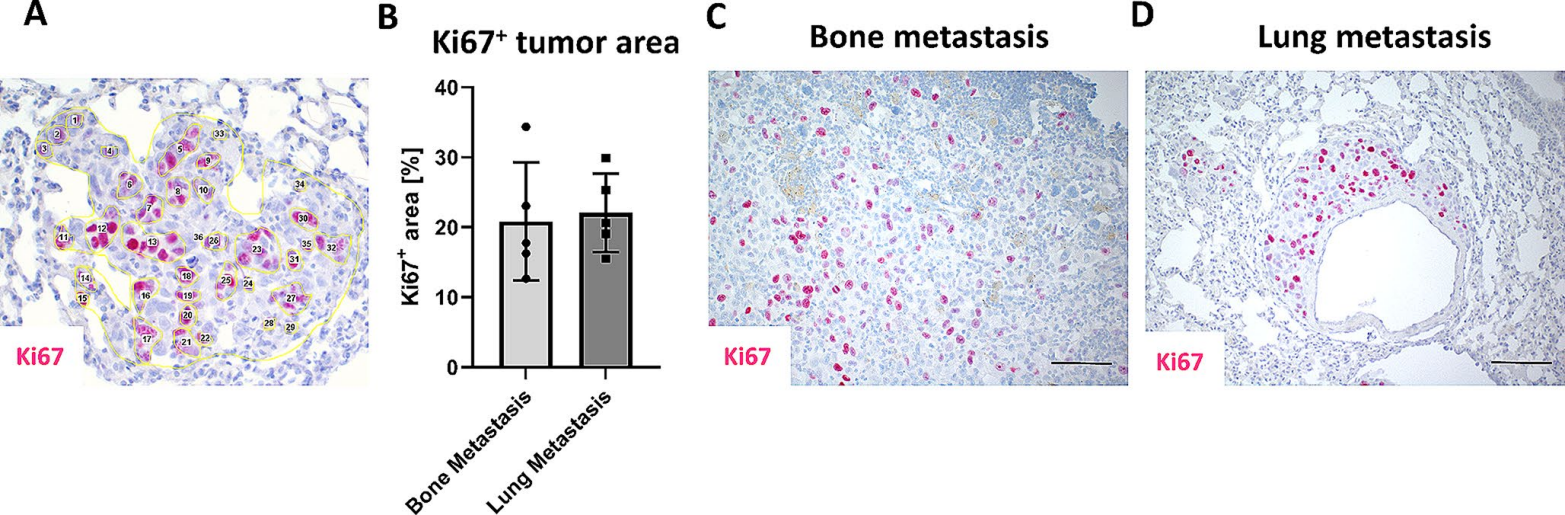
Ki67 analysis of bone and lung metastases. It was interactively drawn around the Ki67^+^ tumor cell area and normalized to the total metastasis area **A** to determine the Ki67 + tumor area / metastasis area [%] shown in (**B**). Representative images of **C** bone and **D** lung metastasis after immunohistochemistry staining against Ki67 are shown. Data show Mean ± SEM, *n* = 5/group, Student’s t-test

In addition to correlation analysis, we performed Bland-Altman (BA) analysis, a statistical method that assesses the agreement between two quantitative methods in order to determine their comparability [22, 23]. Results of BA analysis are described using limits of agreement with corresponding 95% confidence intervals (CI) of these limits and the mean differences between the two measurements. Data are plotted on an XY graph, showing the average of two measures on the X-axis and the difference between these measures on the Y-axis (Figs. 5 and 6). Ideally 95% of all data points should be within ± 1.96 standard deviations from the mean difference and a mean difference of zero between both methods would indicate that the two measurements give exactly the same results [23].

With regards to metastasis quantification in lung samples, the BA analysis showed a mean difference of 0.87 (95% CI: 0.79–0.95, Fig. 5A, B), a lower limit of agreement of 0.10 (95% CI: -0.04 to 0.24, Fig. 5A, B) and an upper limit of agreement of 1.64 (95% CI: 1.50–1.78) when the methods *Alu*-qPCR vs. ex vivo BLI were compared (Fig. 5A). Importantly, the difference between both measurements did not depend on the mean which suggests that the variance across the measurement range is homogenous (Fig. 5A). For *Alu*-qPCR vs. Histo, the mean difference was 3.34 (95% CI: 3.13–3.54, Fig. 5B) with an upper limit of agreement of 4.03 (95% CI: 3.68, upper CI: 4.38, Fig. 5B) and a lower limit of agreement of 2.65 (95% CI: 2.29, upper CI: 3.00; Fig. 5B). For ex vivo BLI vs. Histo, the mean difference was 2.09 (lower CI: 1.87, upper CI: 2.31, Fig. 5C) with an upper limit of agreement of 2.84 (lower CI: 2.46, upper CI: 3.22, Fig. 5C) and a lower limit of agreement of 1.34 (lower CI: 0.96, upper CI: 1.72, Fig. 5C). For *Alu-*qPCR vs. Histo and ex vivo BLI vs. Histo we observed that the differences between both measurements increased with increasing mean (Fig. 5B, C).

With regards to bone metastasis quantification, no comparison between any pair of the methods revealed a distribution in the Bland Altman plot which could be interpreted as agreements with always multiple data points exceeding the limits of agreement and a systematic trend of higher mean values having larger differences between both methods (Fig. 5D–I). This contrasted with the Bland Altman analysis of quantification methods concerning lung metastasis detection.

## Discussion

Metastatic growth at distant organs is initiated by single disseminated tumor cells (DTCs) or small cell clusters that have been shed or actively detached from the primary tumor and ultimately homed to the metastatic site. Once at the metastatic site, the DTCs adapt to a completely different microenvironment [24]. It is well known that DTCs can enter a dormant state or initiate metastatic growth and disease progression at any time, even years after diagnosis and/or successful treatment of the primary tumor. Bone provides a special, supportive environment for prostate DTCs [25]. Tremendous progress has been made in understanding prostate cancer metastasis to bone, however, especially the early steps (homing to bone, awakening from dormancy) of the metastatic cascade remain to be fully elucidated [24]. Given that bone metastasis remains incurable once the tumor is actively proliferating in bone, it is of highest importance to study these events and find suitable targets to prevent or inhibit metastatic outgrowth. For this purpose, it is necessary to precisely detect and quantify DTCs as well as micro- and macro-metastases in the bone microenvironment in preclinical models.

Commonly acknowledged techniques to report bone metastatic burden in long bones of mice include macroscopic imaging techniques (e.g.: X-ray, microcomputed tomography (µCT) and magnetic resonance imaging (MRI)) as well as optical imaging techniques (e.g.: fluorescence, BLI) [26]. In addition, histological examination, flow cytometric and molecular (qPCR) analyses are employed to detect and quantify bone metastases [6, 7, 27]. In particular, ex vivo BLI has been used for detection of micro-metastases in the bone in spontaneous metastasis xenograft models [10, 14, 15]. Here, we assessed the correlation of *Alu-*qPCR, ex vivo BLI and histological examination to determine metastatic load in lung and bone samples and report a considerable discrepancy in bone metastasis quantification between the detection methods, which was not found for lung metastasis quantification.

Bioluminescence (BL) is defined as the light emitted by a living animal through a chemical reaction inside the organism [28]. Briefly, luminescence is generated through the oxidation of luciferin by the enzyme luciferase. Firefly luciferase in particular requires adenosine triphosphate (ATP), oxygen (O_2_) and magnesium (Mg^2+^) as co-factors together with its substrate, D-luciferin [28]. It has been reported that the BLI signal intensity depends on the type, composition and microenvironment of the target organ as well as on the depth of tissue overlying the DTCs in the organ of interest [29]. Depending on the structure and microenvironment of the organ, light absorption, scattering and quenching might occur, thus reducing BLI sensitivity [30, 31]. Bone in particular has a very complex structure consisting of cancellous/trabecular bone and hard, calcified cortical bone that surrounds the marrow cavity in which metastases reside [32]. Consequently, precise BLI signal detection from metastases within the bone might be attenuated compared to the detection of metastases in soft tissues such as the lung where metastatic growth might happen close to or even on the tissue surface.

Furthermore, hemoglobin can hamper the detection of luciferase activity in tissue samples and might thus mask the actual tumor load [33]. Considering that bone is a highly vascularized organ [34] and that hematopoietic cells (e.g. reticulocytes containing hemoglobin) originate from the marrow, this could also be highly relevant for the BLI analysis of long bones and might explain the discrepancy that we observed between the bone metastatic loads obtained by BLI vs. *Alu-*qPCR and histology. Of note, much higher blood flow rates in bone tissue compared to lung tissue have been reported for mice (lung: 0.04 mL/min vs. bone: 0.30 mL/min) [35], which might also affect the accuracy of BLI to determine bone vs. lung metastases. However, blood flow did not take place anymore when BLI scans were made in our ex vivo setup.

It is therefore important to further consider that data acquired from BLI, unlike data from histology or flow cytometry, are not measurements of individual cells but rather a measure of a potentially quite heterogeneous population [26]. In case specific cell subpopulations have varying luciferase expression, this variation might be enhanced in special growth conditions (e.g.: exposure to hypoxia, composition of the tumor microenvironment [36, 37]), where only a highly specialized subpopulation of DTCs might be able to survive and initiate metastatic growth. To this end, we could at least exclude variations in the percentages of Luciferase-positive tumor cells per metastasis in lung vs. bone (observational data only; representative images can be seen in Figs. 2C and 3E). With regards to the presence of oxygen (O_2_), studies have reported decreased BLI signals in cancer cells upon decreased O_2_ concentrations (i.e. hypoxia) [38–40] and we observed similar effects after culturing PC-3 cells in hypoxic conditions (supplemental Fig. 1F). The use of luciferase mutants with bioluminescent properties that enable dual-color reporter assays has therefore been suggested [40]. Bone in particular has been considered a hypoxic environment [41] and might thus, in addition to hypoxic tumor regions, influence the obtained BLI signal intensity [37, 42, 43]. This could be another reason why the quantification of bone metastasis load by BLI is less accurate in bone compared to lung tissues.

As we employed a spontaneous model of metastasis, the metastatic cells that dissociate from the primary tumor will most likely undergo several changes to survive in the bone microenvironment and whether their luciferase expression remains constant during the metastatic process remains to be determined. However, in a parallel study in our group, we did not observe a difference in the in vitro bioluminescence activity of parental PC-3-Luc2/RGB cells when compared to cells that had been isolated from established in vivo lung and bone metastases (manuscript in preparation). Moreover, the activity of firefly luciferase depends on the availability of O_2_ and ATP and thus only metabolically active cells are able to emit photons that can be measured and quantified [44], highlighting that BLI will not capture dead or necrotic tumor areas [43]. Moreover, dormant cells might not be captured by BLI given that dormant cells undergo metabolic adaptations (e.g. reduced metabolism) and thus the luciferase promoter might not be active in these cells. However, lung and bone metastases in our spontaneous metastasis xenograft models contain almost similar percentages of proliferative tumor cells per metastatic lesion as determined by Ki-67 staining (Bone metastasis: 20.81% vs. Lung metastasis: 22.08%, Suppl. Figure 1). Another critical factor is the availability of luciferin as a sufficient amount of substrate should reach the luciferase-expressing cells [26]. How substrate uptake between soft tissues and bone tissues varies, remains to be established. In summary, compared to the detection of lung metastasis the accuracy of BLI to detect bone metastasis might be reduced based on the challenging environmental conditions (i.e. hypoxia, blood flow (haemoglobin) as well as the organ structure (calcified tissue vs. soft tissue).

In addition, it should be considered that the overall level of detectable metastatic cells was much lower in the bones than in the lungs (e.g. 0.241 cells/60 ng DNA up to 1,350 cells/60 ng DNA in the lungs vs. 0.001 tumor cells/60 ng bone marrow DNA up to 65.30 tumor cells/60 ng in the bone marrow as per *Alu*-qPCR). Thus, the aforementioned rather technical and environment-specific limitations of the BLI sensitivity in case of bone metastases might be further complicated by a particularly low cell load at this site. Both aspects might collectively explain the comparably poor correlation of ex vivo BLI with other methods in case of bone metastasis detection. However, we observed a very strong correlation between the BLI^Hi^ and BLI^Lo^ bone (*r* = 0.93) which clearly demonstrates that the method is robust under equal conditions. Considering these drawbacks, we suggest that especially the detection of single DTCs and micro-metastasis in long bones should be carried out with additional and/or multiple techniques.

We performed *Alu*-qPCR of lung and bone marrow samples and could reliably detect metastases in 100% and 71.62% of samples, respectively. Similarly, others have also reported (*Alu-*) qPCR as the most reliable method for the detection of smallest metastatic burden in xenograft models of bone metastasis [45]. With a length of about 300 nucleotides, *Alu* elements are primate-specific short interspersed elements (SINEs) in the human genome [46]. As these sequences are not present in rodents, they enable a precise detection of human tumor cells in xenograft models. However, this analysis has to be performed ex vivo and the assessed tissue is destroyed making the parallel analysis of histology impossible.

Histological analysis such as H&E or IHC staining for tumor cell-specific markers (e.g. luciferase) enables a precise tumor cell localization and analysis of the pathophysiological alterations occurring during the establishment of bone metastases as well as an in-depth analysis of the interactions between DTCs and cells of the bone microenvironment [47, 48]. However, the detection of single DTCs within the bone microenvironment might be challenging, especially when not using a specific marker that can be detected by IHC [49]. It might be speculated that the incidence of bone metastases determined by histology differs from data obtained by other methods because of a too small number of histological slides that has been assessed. However, here we assessed an extensive amount of slides per bone sample as we cut serial sections of bones and evaluated every 10th (H&E-stained) and 20th section (Luciferase-stained) for the presence of single DTCs, micro- and macro-metastases, resulting in an average of 25 to 35 individual luciferase-stained bone sections per mouse, covering a tissue depth of 125 to 175 μm. Nevertheless, we could only detect (luciferase-stained) tumor cells in 18.67% of analyzed mice (n total = 75, Table 1). In order to assess the whole bone by histology, improved quantification approaches such as thicker sections and advanced imaging methodologies, ideally whole bone imaging (i.e. tissue clearing), would be required. In agreement with our study, where we obtained a discrepancy in the correlation of *Alu-*qPCR, histology and ex vivo BLI for the quantification of bone metastases, also others have reported a discrepancy of flow cytometry, qPCR and immunohistochemistry/ histology regarding their specificity to detect low metastatic burden in the bone [49]. However, it has to be considered that our study is limited by the use of one cell line only.

In summary, many techniques can be used for the quantification of (bone) metastases. However, our study highlights that results might vary based on the employed analysis method. Consequently, the choice of technique to detect and/or monitor bone metastases should be selected critically and will have to depend on the study design (i.e. route of tumor cell inoculation, cell line, specific and stable marker expression etc.). Of note - although outside the scope of this manuscript - also the immune status of the mouse model should be considered given that immune competent mouse models could be immunologically intolerant to reporter proteins (e.g. luciferase or fluorescence proteins) [50–53]. Our results highlight that although the detection methods for lung metastasis appear to be interchangeable the analysis of bone metastasis might require a combination of at least two techniques (Supplemental Fig. 1D, E). Based on our experiments we would consider histological analysis after IHC staining for a marker that is specifically expressed by the used cell line essential for an accurate analysis of bone metastatic burden and suggest the use of IHC in combination with Alu-qPCR, BLI or alternative methods such as flow cytometry (Supplemental Fig. 1D, E.). Especially for the detection and quantification of single DTCs and micro-metastases in bone, BLI alone - at least in the ex vivo setup - appears to be insufficient to precisely determine the bone metastatic load while it is suitable for the quantification of lung metastases. It remains to be determined whether similar drawbacks exist for bone metastasis quantification based on in vivo BLI. Novel techniques are developed including *intra-vital* microscopy of live animals using multiphoton microscopy [54] or bone clearing which allows three-dimensional imaging of the whole bone without sectioning the tissue [55] and will hopefully facilitate studies of bone metastasis.

### Electronic supplementary material

Below is the link to the electronic supplementary material.

**Supplemental Figure 1:** A limiting dilution curve of the detection limits for (A) Bioluminescence Imaging (BLI), (B) Alu-qPCR and (C) Immunohistochemical staining (IHC). (A) PC-3 cells were plated in concentrations from 1000 - 1 cells/well into a 96-well plate and bioluminescence signal intensity measured (total flux [p/s]). (B) Alu-qPCR standard curve containing serial dilutions ranging from 2000-0.002 human tumor cells per mouse DNA template (60ng total DNA).≤ (C) Luciferase positive tumor cells can be detected even as single cells in the bone using IHC. Recommendations on the use of quantification methods for lung (D) and (E) bone metastases. (F) Luciferase activity (total flux in photons per second [p/s]) of PC-3 RGB cells after 72 hrs and 96 hrs in hypoxic (37 °C; 5% CO_2_, 1% O_2_) or normoxic (37 °C; 5% CO_2_, 20% O_2_) conditions. Unpaired t-test with ** is p ? 0.01 and **** is p ? 0.0001.

## Data Availability

All data generated or analysed during this study are included in this published article and its supplementary information files.

## Abbreviations

ATP: Adenosine triphosphate
BA: Bland-Altman
BLI: Bioluminescence imaging
CI: Confidence interval
DTC: Disseminated tumor cell
EDTA: Ethylenediaminetetraacetic acid
EMT: Epithelial-mesenchymal transition
H&E: Hematoxylin and eosin
Hi: High
hrs: Hours
IHC: Immunohistochemistry
Lo: Low
Luc, Luc2: Luciferase
MET: Mesenchymal-epithelial transition
Mg^2+^: Magnesium
mL: Milliliter
min: Minutes
O_2_: Oxygen
p/s: Photons per second
PCa: Prostate cancer
qPCR: Quantitative polymerase chain reaction
s.c: Subcutaneous
