# Supplementary figures and images for: Comparison of ex vivo bioluminescence imaging, Alu-qPCR and histology for the quantification of spontaneous lung and bone metastases in subcutaneous xenograft mouse models
Source: Clin Exp Metastasis. 2024 Feb 14;41(2):103–15. doi: 10.1007/s10585-024-10268-4 (PMC10972982; doi:10.1007/s10585-024-10268-4)

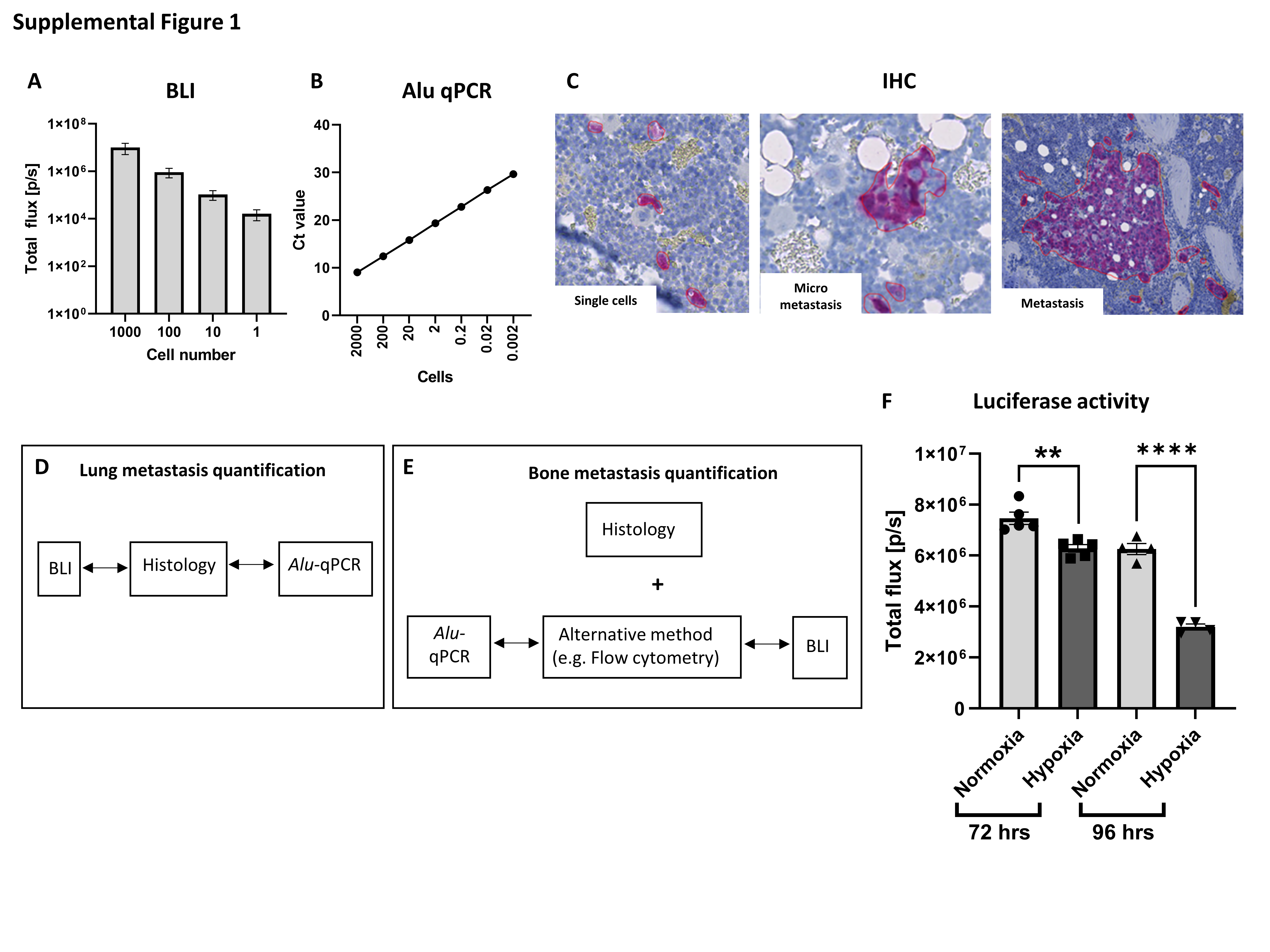

Supplement: Supplementary file 1 — Supplemental Figure 1: A limiting dilution curve of the detection limits for (A) Bioluminescence Imaging (BLI), (B) Alu-qPCR and (C) Immunohistochemical staining (IHC). (A) PC-3 cells were plated in concentrations from 1000 - 1 cells/well into a 96-well plate and bioluminescence signal intensity measured (total flux [p/s]). (B) Alu-qPCR standard curve containing serial dilutions ranging from 2000-0.002 human tumor cells per mouse DNA template (60ng total DNA).≤ (C) Luciferase positive tumor cells can be detected even as single cells in the bone using IHC. Recommendations on the use of quantification methods for lung (D) and (E) bone metastases. (F) Luciferase activity (total flux in photons per second [p/s]) of PC-3 RGB cells after 72 hrs and 96 hrs in hypoxic (37 °C; 5% CO2, 1% O2) or normoxic (37 °C; 5% CO2, 20% O2) conditions. Unpaired t-test with ** is p ? 0.01 and **** is p ? 0.0001. [file 10585_2024_10268_MOESM1_ESM.tif]
